# Detection and Characterization of *Cucumis melo* Cryptic Virus, *Cucumis melo* Amalgavirus 1, and Melon Necrotic Spot Virus in *Cucumis melo*

**DOI:** 10.3390/v11010081

**Published:** 2019-01-18

**Authors:** Binhui Zhan, Mengji Cao, Kaina Wang, Xifeng Wang, Xueping Zhou

**Affiliations:** 1 State Key Laboratory for Biology of Plant Disease and Insect Pest, Institute of Plant Protection, Academy of Agricultural Sciences, Beijing 100193, China; binhuizhan@126.com (B.Z.); kainawang@163.com (K.W.); xfwang@ippcaas.cn (X.W.); 2 National Citrus Engineering Research Center, Citrus Research Institute, Southwest University, Chongqing 400715, China; caomengji@cric.cn; 3 State Key Laboratory for Rice Biology, Institute of Biotechnology, Zhejiang University, Hangzhou 310058, China

**Keywords:** melon (*Cucumis melo*), transcriptome sequencing, dsRNA virus, *Amalgavirus*, *Deltapartitivirus*

## Abstract

Three RNA viruses—*Cucumis melo* cryptic virus (CmCV), *Cucumis melo* amalgavirus 1 (CmAV1), and melon necrotic spot virus (MNSV)—were identified from a melon (*Cucumis melo*) transcriptome dataset. CmCV has two dsRNA genome segments; dsRNA-1 is 1592 bp in size, containing a conserved RNA-dependent RNA polymerase (RdRp), and dsRNA-2 is 1715 bp in size, and encodes a coat protein (CP). The sequence alignment and phylogenetic analyses of the CmCV RdRp and CP indicated CmCV clusters with approved or putative deltapartitiviruses in well-supported monophyletic clade. The RdRp of CmCV shared an amino acid sequence identity of 60.7% with the closest RdRp of beet cryptic virus 3, and is <57% identical to other partitiviruses. CmAV1 is a nonsegmented dsRNA virus with a genome of 3424 bp, including two partially overlapping open reading frames (ORFs) encoding a putative CP and RdRp. The sequence alignment and phylogenetic analyses of CmAV1 RdRp revealed that it belongs to the genus *Amalgavirus* in the family *Amalgaviridae*. The RdRp of CmAV1 shares 57.7% of its amino acid sequence identity with the most closely related RdRp of *Phalaenopsis equestris* amalgavirus 1, and is <47% identical to the other reported amalgaviruses. These analyses suggest that CmCV and CmAV1 are novel species in the genera *Amalgavirus* and *Deltapartitivirus*, respectively. These findings enrich our understanding of new plant dsRNA virus species.

## 1. Introduction

Melon (*Cucumis melo*) is an economically important fruit crop in many areas of the world. China was the main producer of melon in 2016, with approximately 38.1% of the world’s cultivating area and 51.1% of total crop production (FAOSTAT, http://faostat.fao.org). However, the yield and quality of melon has been increasingly affected by the incidence and severity of diseases. Viral diseases are one of the most common constraints in melon production among the known pathogen-induced diseases. There are at least 45 characterized viruses that have been reported infecting melon worldwide [1]. In China, the major viruses that can cause serious damages in melon production are cucurbit aphid-borne yellow virus, zucchini yellow mosaic virus, watermelon mosaic virus, tobacco mosaic virus, cucumber mosaic virus, and squash mosaic virus, among others [1,2].

The *Partitiviridae* is a family of dsRNA viruses with bisegmented genomes that are 3–4.8 k base pairs (bp) in length [3,4]. The two genome segments are encapsidated individually. Each dsRNA contains one open reading frame (ORF), with one encoding the RNA-dependent RNA polymerase (RdRp) and the other encoding the coat protein (CP) [3]. The *Partitiviridae* family is classified into five genera, with characteristic hosts for the members of each genus. The genera *Alphapartitivirus* and *Betapartitivirus* include viruses isolated from either fungi or plants, whereas the genus *Gammapartitivirus*, so far, contains viruses isolated only from fungi, and the genus *Deltapartitivirus*, to date, comprises viruses isolated only from plants [3,4]. The genus *Cryspovirus* contains one member, which was isolated from a protozoan [4]. There are also 15 species in this family that are yet to be assigned to a genus. Besides the characteristic hosts within each genus, genome segments, and proteins lengths, RdRp amino acid sequence identity and phylogenetic grouping are also included in the genus demarcation criteria within the family *Partitiviridae* [3]. Partitiviruses are generally associated with latent infections, or with symptoms that are mild and difficult to detect [5]. Recently, some novel partitiviruses that influence the physiology of the host fungi and symptom of plants were reported [6,7,8,9]. Partitiviruses are transmitted with high efficiency intracellularly via the seeds of plants, the oocysts of protozoa or hyphal anastomosis, cell division, and the sporogenesis of fungi [3]. Transmission is not possible by mechanical inoculation or grafting, and there are no known natural vectors [3].

*Amalgaviridae* is a recently recognized family of double-stranded RNA viruses that contains only one genus, *Amalgavirus*, which includes four species of plant viruses (*Blueberry latent virus*, *Rhododendron virus A*, *Southern tomato virus*, and *Vicia cryptic virus M*) according to ICTV taxonomy (https://talk.ictvonline.org/taxonomy/). These plant amalgaviruses are monopartite, and have small dsRNA genomes of ~3.5 kbp in length containing two partially overlapping open reading frames (ORFs). ORF1 encodes products with uncertain functions that bare similarity to the nucleocapsid protein [10] and replication factory matrix-like protein [11]. The downstream ORF2 encodes an RdRp, which is predicted to be expressed via a +1 programmed ribosomal frameshifting (PRF) event during the translation of ORF1 [12]. The +1 PRF motif sequence UUU_CGN (underline indicates the codon boundary for ORF1; N, any nucleotide), is prevalent in most amalgaviruses [12,13]. When it occurs, the +1 PRF, the codon frame changes from UUU_ CGN (ORF1) to U_UUC_GNN (ORF2) [12]. The same +1 PRF motif is observed in other RNA viruses such as *Zygosaccharomyces bailii* virus Z (ZbV-Z) and influenza A viruses [14,15]. The amalgaviruses possess characteristics of both totiviruses and partitiviruses, with the genome architecture of *Totiviridae* and gene evolutionary resemblance to *Partitiviridae*, indicating a likely genetic relation to these two families [10,16]. Members of this family are transmitted vertically via seeds, and are not thought to be capable of efficient extracellular transmission, unless mediated by an unknown vector [17,18].

Melon necrotic spots virus (MNSV) is a single-strand positive RNA virus, which is in the *Gammacarmovirus* of the family *Tombusviridae*. It is naturally transmitted by seed with the assist of fungus, and can be readily mechanically transmitted to several species of *Cucurbitaceae* [19,20]. The virus causes a significant decrease in the yield in melon, and induces necrotic lesions on inoculated leaves [21].

During the exploitation of nucleic acid sequences of viruses, multiple gene expression mechanisms were used by viruses to compress maximal information into limited genome space. For example, an overlapping essential P3-PIPO protein was translated in the embedded P3 cistron with +2 PRF, which is prevalent in the family *Potyviridae* apart from the mechanism of large polyprotein [22]; in addition to ribosome shunting, RNA splicing was adopted by the cauliflower mosaic virus to generate several isoforms of 35S RNA for the benefit of regulating the expression of ORFs [23]; readthrough and RNA recombination were identified in the analyses of turnip crinkle virus [24] and tomato bushy blunt virus [25], etc. In this article, we discovered two dsRNA viruses and one ssRNA virus. In the course of the investigation of viral genomes, we recovered additional instances of such multiple uses. Frameshift and RNA recombination simultaneously existed in the expression of the *Cucumis melo* amalgavirus 1 (CmAV1) genome.

## 2. Materials and Methods

### 2.1. Sample Collection and RNA Sequencing

In July 2017, leaf samples with yellow vein symptoms were collected from melon plants grown in the greenhouse in Heilongjiang Province, China. Samples were stored at −80 °C.

The symptomatic leaves were used for total RNA extraction with TRIzol Reagent (Invitrogen, Carlsbad, CA, USA) following the manufacturer’s instructions. The Ribo-Zero^TM^ rRNA removal kit (plant leaf) (Epicentre, Madison, WI, USA) was selected to deplete ribosomal RNA from the total RNA. The prepared ribo-depleted RNA sample was used to construct a cDNA library with TruSeq RNA Sample Prep Kit v2 (Illumina, San Diego, CA, USA), which then was subjected to next-generation sequencing (NGS) using the Illumina HiSeq 4000 platform with a paired-end 150 setup (Mega Genomics, Beijing, China). The raw reads from the NGS were initially processed by adapter trimming, quality trimming, and length trimming through CLC Genomics Workbench 9.5 (Qiagen, Valencia, CA, USA) to generate clean reads. To detect unknown viruses in symptomatic melon, the clean reads were mapped to the reference assembly genome of *C. melon* cv. DHL92 (NCBI GenBank assembly accession: GCA_000313045.1) for exclusion, while the unmapped reads were extracted for further analysis. CLC Genomics Workbench 9.5 was used for the de novo assembly of contigs, which were subsequently used as queries for BLAST searches against the NCBI databases.

### 2.2. RACE Analysis and Validation of the Full Genomes

To verify the presence of viruses in the symptomatic leaves, we used reverse transcription PCR (RT-PCR) to detect and amplify the sequences of the putative viruses. The 5’-terminal and 3′-terminal sequence of the genomic RNAs were determined through 5′RACE and 3′RACE systems using a SMARTer RACE cDNA Amplification Kit (Clontech, Mountain View, CA, USA) according to the manufacturer’s instructions. The specific primers for RT-PCR and 5′ gene specific primers (GSPs) and 3′ GSPs for 5′RACE and 3′RACE, respectively, were designed according to the assembled contigs (listed in Table S1). The PCR products were purified with an E.Z.N.A. Gel Extraction Kit (Omega, Norcross, GA, USA), and then cloned into a pEASY-T-Blunt vector (TransGen Biotech, Beijing, China). The recombinant clones were sequenced with universal primer pairs M13F/M13R via Sanger sequencing (TsingKe Biotech Co., Beijing, China). More than five independent clones were sequenced for each fragment.

### 2.3. Sequence and Bioinformatics Analyses

Sequences were analyzed and assembled with the software DNAMAN version 5.0 (Lynnon Biosoft, Quebec, QC, Canada) and then the assembled whole sequences were submitted to the GenBank database in NCBI using the WWW-based submission tool BankIt. The following bioinformatics analyses were performed with the new viral genomes. The ORF Finder program (http://www.ncbi.nlm.nih.gov/projects/gorf/) in NCBI was used for ORF prediction. The multiple sequence alignment and conserved domains prediction of the RdRp were performed by CLC Genomics Workbench 9.5. The RNA secondary structure was predicted with the online prediction tool RNAstructure (http://rna.urmc.rochester.edu/RNAstructureWeb/). Protein structure prediction was conducted with the Garnier program (http://www.bioinformatics.nl/cgi-bin/emboss/garnier) and Quick2D (https://toolkit.tuebingen.mpg.de/#/tools/quick2d). The ClustalW method was applied to multiple sequence alignments, and MEGA 7.0 [26] was used for phylogenetic tree construction with the neighbor-joining method with 1000 bootstrap replicates, Poisson model, and pairwise gap deletion options. The sequence pair identities were calculated and aligned with the MAFFT program (https://www.ebi.ac.uk/Tools/msa/mafft/); then, Sequence Demarcation Tool (SDT) software displayed pairwise identity scores using a color-coded matrix [27].

## 3. Results

### 3.1. Identification of Three RNA Viruses in Melon Using Next-Generation Sequencing

**Figure 1:**
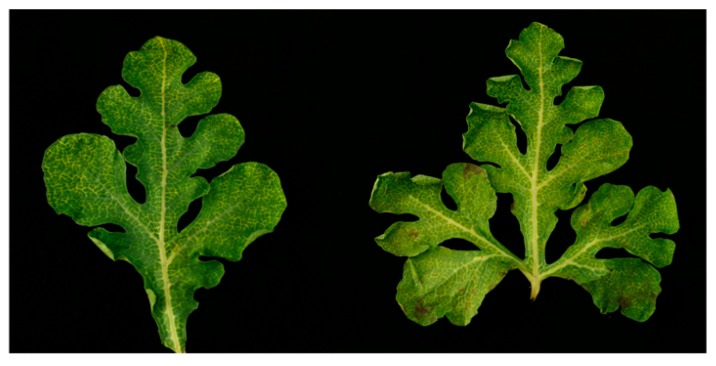
Symptoms of the melon leaves showing yellow vein in Heilongjiang Province in China.

In July 2017, the melon plants showing severely yellow vein symptoms were observed in a greenhouse in Heilongjiang Province in China, and the leaves from different plants with classical symptoms were collected and photographed (Figure 1). To identify the viruses present in the melon samples, RNAs were isolated from three different leaves and subjected to RNA-seq together. For RNA-seq, a total of 83,348,684 clean reads were obtained after adapter trimming, quality trimming, and length trimming, and after filtering the reads mapping to the melon genome. After mapping with the melon genome, the unmapped reads were de novo assembled into contigs. The results of BLASTn, using the obtained contigs as queries, suggested that three kinds of RNA viruses exist in the symptomatic melon transcriptome, as shown in Table 1. Most of the viral reads were from partitivirus dsRNA-1 and dsRNA-2, with an average coverage of reference genomes of 1076.38 and 2611.19 times, respectively. Conversely, other reads corresponded to an amalgavirus, with the average coverage of 68.92 times, and MNSV, with 88.57 times coverage.

RT-PCR amplification with the specific primer pairs (Table S1) and sequencing of the fragments confirmed the presence of these contigs and determined the viral sequences in three detected leaves from different plants. In addition, the 5′-terminus and 3′-terminus of the partitivirus and amalgavirus were amplified and sequenced with 5′RACE and 3′RACE systems. To obtain the complete viral genomic RNAs, sequences of several fragments covering the whole genome were assembled with DNAMAN software.

### 3.2. Characterization of CmCV

**Figure 2:**
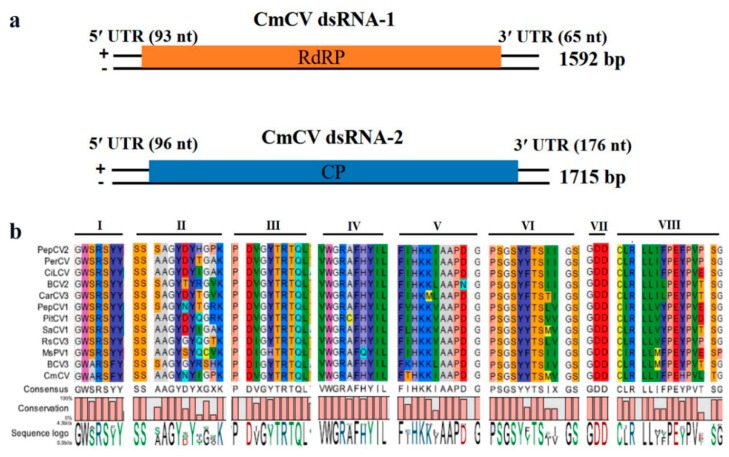
Schematic representation of the genome organizations of *Cucumis melo* cryptic virus (CmCV) (**a**) and amino acid alignment (**b**) between conserved RNA-dependent RNA polymerase (RdRp) motifs of CmCV and selected proved or putative deltapartitiviruses.

The partitivirus isolated from *C. melon* was named *Cucumis melo* cryptic virus (CmCV). The whole genome of the CmCV consisted of two dsRNA segments, dsRNA-1 with a length of 1592 bp (NCBI GenBank No. MH479772) and dsRNA-2 (NCBI GenBank No. MH479773) with a length of 1715 bp (Figure 2a). Similar to the other partitiviruses, each of these two dsRNA segments contained one ORF. The ORF1 in dsRNA-1 starts at 94 nt, and ends with 1527 nt with a TAA stop codon, encoding a protein of 477 amino acids (aa) with an estimated molecular weight of ~54.6 kDa. A conserved RdRp domain (pfam00680) from aa positions 190 to 400 was detected in the protein. The protein sequence alignments showed that it shares the highest identity of 60.1% with the predicted RdRp of beet cryptic virus 3 (BCV3), which is in the *Deltapartitiviruses* genus of the *Partitiviridae* family. It also shows significant similarities with the RdRp of other definitive or putative deltapartitiviruses, such as with pepper cryptic virus 2 (PepCV2) with an identity of 58.5%, beet cryptic virus 2 (BCV2) with an identity of 57.2%, carnation cryptic virus 3 (CarCV3) with an identity of 57.0%, and persimmon cryptic virus (PerCV) with an identity of 58.4%, among others. Furthermore, multi-alignments of RdRps with high similarities using CLC Genomics Workbench 9.5 revealed that CmCV RdRp contains several conserved motifs of the deltapartitiviruses, such as motif I “GWSRSYY” and motif III “PDVGYTRTQL” (Figure 2b). The ORF2 spanning 97 nt to 1539 nt in CmCV dsRNA-2 encoded a CP of 480 aa of ~54.5 kDa. The protein sequence alignments showed that it shares the highest identity of 30.2% with the predicted CP of pittosporum cryptic virus 1 (PitCV1) (Table S2) and subsequently with *Sinapis alba* cryptic virus 1 (SaCV) of 28.1%, *Citrullus lanatus* cryptic virus (CiLCV) of 27.9%, CarCV3 of 27.8%, pepper cryptic virus 1 (PepCV1) of 26.1%, etc.

**Figure 3:**
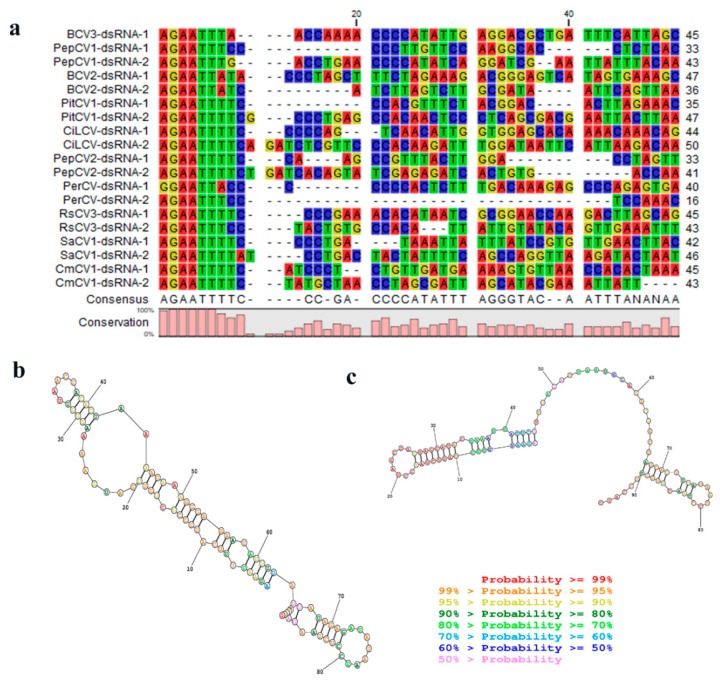
Terminal characteristics of the CmCV dsRNA-1 and dsRNA-2. (**a**) The conserved nucleotide stretches are present at the 5′-termini of CmCV dsRNA-1 and dsRNA-2. The secondary structure formed by the 5′-untranslated regions (5′UTR) of CmCV dsRNA-1 (**b**) and dsRNA-2 (**c**).

Many dsRNA viruses have conserved 5′-terminal and 3′-terminal sequences, which are generally thought to be involved in RdRp recognition or RNA packaging. The 5′-untranslated and 3′-untranslated regions (5′UTR and 3′UTR) of CmCV dsRNA-1 are 93 nts and 65 nts in length. The UTRs of dsRNA-2 are 96 nts and 176 nts in length, respectively. A 5′-AGAATTTTC-3′ sequence was found at the 5′-terminus in both the dsRNA-1 and dsRNA-2 of CmCV, which is highly conserved in deltaparititiviruses (Figure 3a). Additionally, an in silico analysis of the secondary structures of the 5′UTRs of dsRNA-1 and dsRNA-2 using Mathews RNAstructure predicted stable stem-loop structures in these regions (Figure 3b,c).

**Figure 4:**
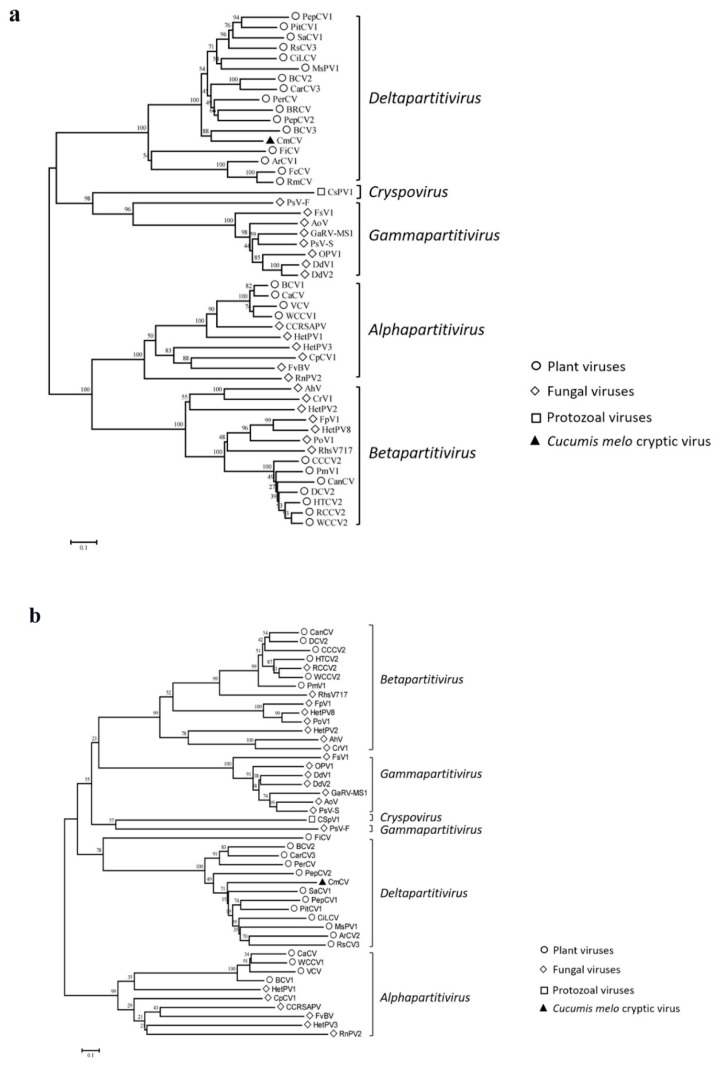
Phylogenetic relationships of CmCV and other members of the family *Partitiviridae* based on RdRp (**a**) and coat protein (CP) (**b**) amino acid sequences. The phylogenetic trees were constructed using the neighbor-joining method by MEGA 7.0 with a bootstrap of 1000 replicates. The symbols indicate the viruses isolated from the same group of host, such as plants, fungi, and protozoa.

In addition, multiple alignments of RdRp and CP amino acid sequences were performed using the ClustalW; then, phylogenetic trees were constructed using the neighbor-joining algorithm based on the alignments of the viruses of all five genera in the family *Partitiviridae* with a bootstrap of 1000 replicates in MEGA 7.0. All of these viruses fell into five phylogenetic clades, in accordance with five known taxonomic genera in the family *Partitiviridae*, as shown in Figure 4a. Based on RdRp alignments, our newly identified CmCV clustered with BCV3 within a monophyletic clade that included other approved or putative deltapartitiviruses. Similar grouping was also observed when phylogenetic analysis was performed based on the CP comparisons (Figure 4b).

To further determine the classification status of CmCV, pairwise alignments of the CmCV RdRp and CP with other partitiviruses were conducted with DNAMAN software. The sequence identities of CmCV RdRp with alphapartitiviruses were between 12.4–16.9%; the sequence identities of CmCV RdRp with betapartitiviruses were <12.6%; the sequence identities of CmCV RdRp with garmmapartitiviruses were between 15.8–18.0%; and the sequence identity of CmCV RdRp with the cryspovirus was 16.4%. Further comparisons with approved and unclassified putative deltapartitiviruses revealed identities between 34.6–60.1%. Thus, the sequence identities of CmCV CP with the approved and putative deltapartitiviruses ranged from 21.9% to 30.1% (except with the fig cryptic virus, which had a sequence identity of 9.9%). However, the identities were no more than 13.2% with the viruses of the other four genera (the identities are shown in Table S2).

**Figure 5:**
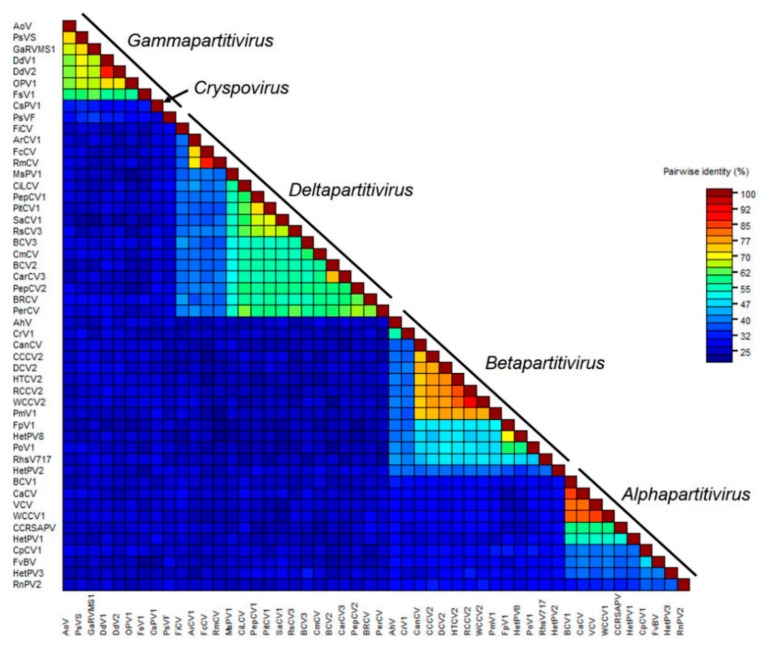
The pairwise identities plot of RdRps in the family of *Partitiviridae* aligned by MAFFT and displayed by Sequence Demarcation Tool (SDT) software.

The SDT software was used to display the pairwise identity scores of RdRps between all of the partitiviruses aligned by the MAFFT program. The color-coded matrix plot showed that pairwise identities can be divided into five groups, which are generally consistent with the five genera classification in the family (Figure 5). The within-genus pairwise identity scores were relatively high, for example, from 32.0% to 84.9% in the *Alphapartitivirus*, from 33.4% to 90.2% in the *Betapartitivirus*, from 35.2% to 87.9% in the *Deltapartitivirus*, and from 27.3% to 87.6% in the *Gammapartitivirus*. In contrast, most of the pairwise identity scores between-genera were <30%. CmCV was positioned in the deltapartitiviruses group, with higher identity scores ranging from 37.4% to 60.7% (Table S3) (a minor difference with DNAMAN calculated with a different algorithm).

Based on the phylogenetic results described above and the other findings, CmCV exhibited many characteristics of the deltapartitiviruses. Consequently, we suggest that it be considered a candidate new species in the genus *Deltapartitivirus*.

### 3.3. Characterization of CmAV1

**Figure 6:**
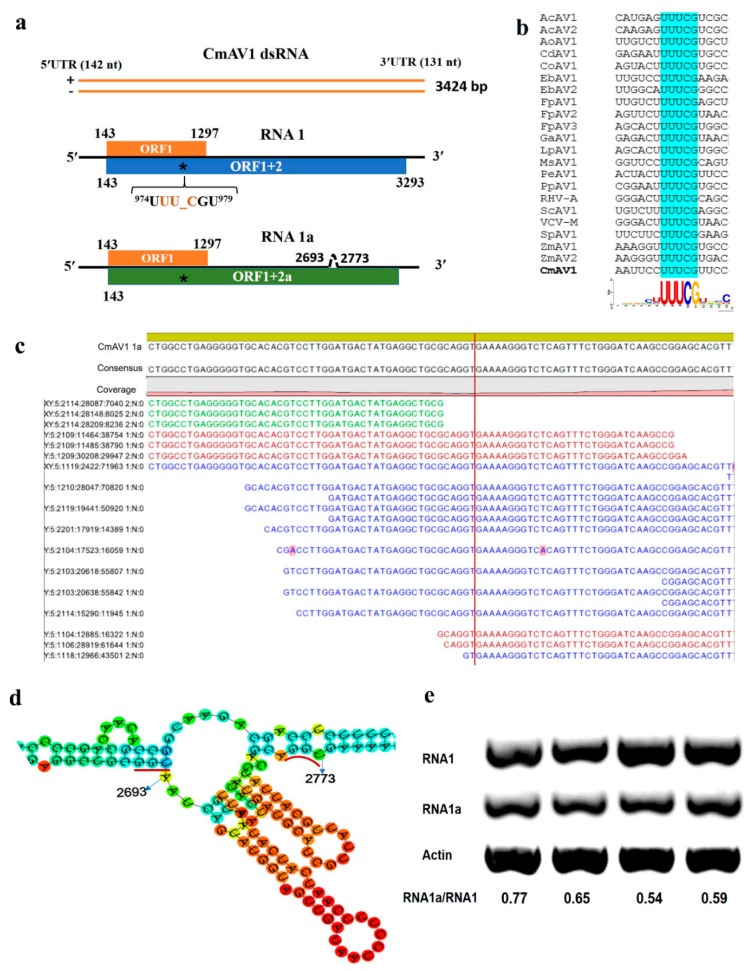
Schematic representation of the genome organizations of *Cucumis melo* amalgavirus 1 (CmAV1) (**a**) and the putative +1 programmed ribosomal frameshifting motif of CmAV1 (**b**). A sequence logo is presented at the bottom. (**c**) The segments that match the junction regions existed in the transcriptome dataset. (**d**) RNA structure of the deleted 2693–2773 region of RNA1 predicted by RNAfold in silico contained two hairpin structures. (**e**) The ratio of RNA1a to RNA1 in four leaves from different plants.

The amalgavirus isolated from *C*. *melo* was named CmAV1. The determined genome sequence of CmAV1 (NCBI GenBank No. MH479774) was 3424 bp in length and contained two partially overlapping ORFs in its positive strand (Figure 6a). The ORF1 starts at 143 nt and ends at 1297 nt, encoding a putative CP of 384 aa with a calculated molecular weight of ~43.0 kDa. No significant matches were found when this protein was compared with representatives in the pfam database. Prediction of the three-dimensional (3D) structure with the EMBOSS tool Garnier and secondary structure overview with Quick2D revealed that there was typical α-helix in the N-terminal position 29–159 aa of this protein, as observed in all of the different proposed and approved plant amalgaviruses. Aside from the common feature of the high content of the predicted α-helix, the amalgaviral CPs are highly divergent, and share only 19–25% sequence identities. The pairwise comparisons of the putative CmAV1 CP with other amalgaviral CPs were performed with DNAMAN software, which showed that most pairwise identities were <23%, except for *Phalaenopsis equestris* amalgavirus 1 (PeAV1), where it was 41.9% (Table S4).

The second protein is predicted to be an ORF1+2 fusion product that would be produced by a +1 PRF mechanism, which is prevalent in amalgaviruses. The CmAV1 genome sequence was predicted to have a putative +1 PRF sequence: ^974^UUU CGU^979^. The predicted ORF2 of CmAV1 started at the nucleotide position 975, which is the first base after the +1 PRF site (Figure 6b). The ORF1 + 2 in CmAV1, spanning position 143–973 nt and 975–3293 nt, encoded a 1049 aa protein in length, and had an estimated molecular mass of ~119 kDa. However, during the amplification and sequencing of the complete sequence of CmAV1, we found that the segments corresponding to 2693–2773 were deleted in 3/10 of the sequenced clones. The transcriptome dataset also showed that there existed segments matching to the junction regions (Figure 6c). The RNA sequence with 2693–2773 deleted was named RNA1a (Figure 6a). Sequence analyses indicated that RNA1a encodes an ORF1+2a with the same reading frame of ORF1 + 2, which expresses a shorter RdRp of ~115 kDa without an important conserved domain (MVVDNPXNHHNVHH) among amalgaviruses. The prediction of RNA1 in silico was conducted by the RNAfold webserver, which showed that the 2693–2773 region contained two hairpin structures (Figure 6d). Moreover, GC-rich and AU-rich sequences were located in the upstream and downstream of the hairpin structures. These characteristics prompted us to speculate that the shorter RNA1a may be produced by replicases hopping over RNA1 through a recombination mechanism. Semi-quantitive RT-PCR with primers specific to RNA1 and RNA1a was conducted with four leaves from different plants to verify the RNA1a production and estimate the ratio between RNA1a and RNA1. The amplification of *Actin* cDNA was used as an internal control. Figure 6e showed that the amount of RNA1a accumulated to a detectable level, and the ratios of RNA1a to RNA1 were 0.54–0.77 in four leaves from different plants.

The 5′UTR and 3′UTR of the genome are 142 and 131 nts long, rich in AU (58.5% and 60.3%, respectively), and are capable of forming complex secondary stem loop-like structures (Figure S1), which might assist RdRp recognition in the process of virus replication.

**Figure 7:**
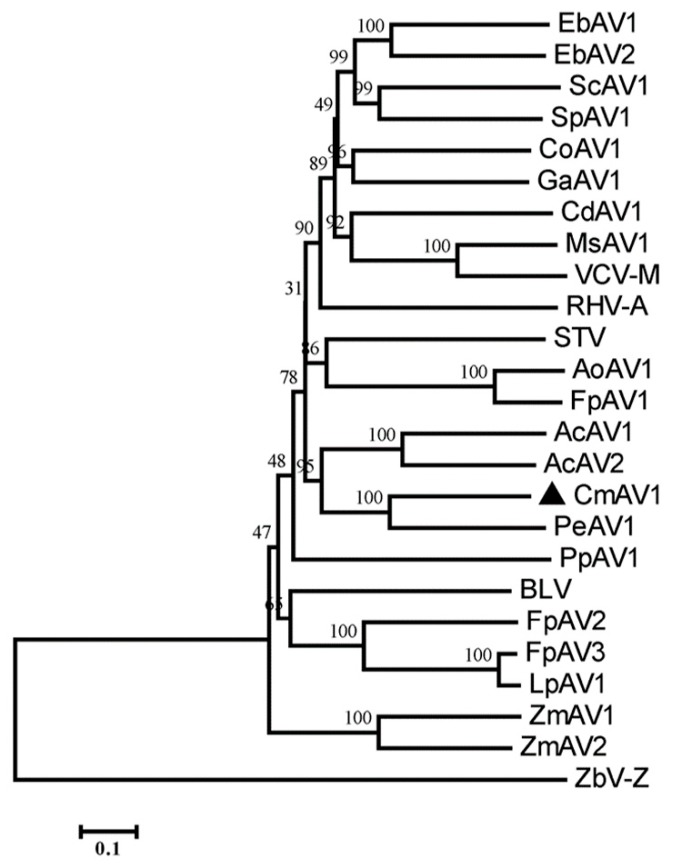
Phylogenetic relationships of CmAV1 and other members of the family *Amalgaviridae* based on RdRp amino acid sequences.

**Figure 8:**
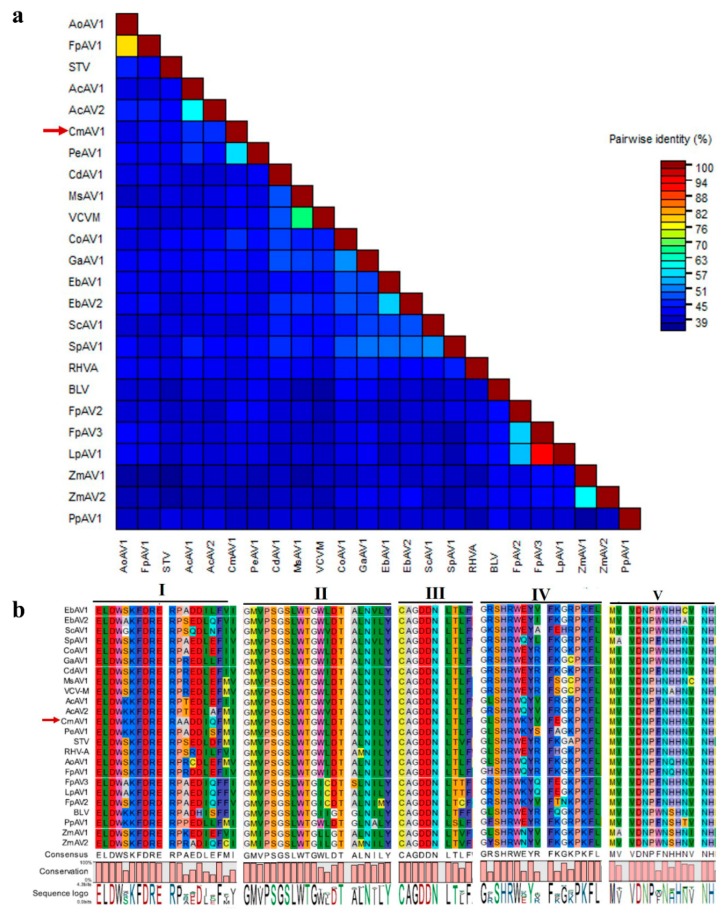
The sequence similarities of RdRps in the family *Amalgaviridae*. (**a**) The pairwise identities plot of RdRps in the family of *Amalgaviridae* aligned by MAFFT and displayed by SDT software. (**b**) Amino acid alignment between conserved RdRp motifs of CmAV1 and the selected amalgaviruses.

The ORF1+2 protein sequences of CmAV1, and other amalgaviruses, were multiply aligned using ClustalW. A phylogenetic tree was constructed by the neighbor-joining method using the MEGA 7.0 software, which indicated that CmAV1 is in a clade with approved and putative amalgaviruses, but in a different clade than ZbV-Z, which is the type species of the fungus-infecting genus *Zybavirus* (Figure 7). Within the amalgaviruses cluster, CmAV1 is more closely related to PeAV1 from *Phalaenopsis equestris*. The pairwise sequence identities conducted by MAFFT and SDT software showed that the ORF1+2 protein of CmAV1 shared 38.5–57.7% aa sequence identities with the other amalgaviruses, and showed the highest identity with PeAV1: 57.7% (Figure 8a and Table S5). Furthermore, multiple alignments of RdRps with higher similarities using CLC Genomics Workbench 9.5 revealed that RdRp contains several conserved motifs, as shown in Figure 8b.

## 4. Discussion

The development of RNA NGS and bioinformatics has accelerated the discovery of novel viruses [28,29,30]. Through the analyses of transcriptome data, many dsRNA viruses have been identified and characterized recently, such as the two novel amalgaviruses from the *Zostera marina* [13], a novel alphapartitivirus *Raphanus sativus* cryptic virus 4 from radish [31], the *Pyrus pyrifolia* cryptic virus from Japanese pear [32], and the CiLCV infecting watermelon [2], among others. Furthermore, Nibert et al. have demonstrated that 19 accessions from the transcriptome shotgun assembly database encompass the amalgaviruses [12]; this substantially expands the members of *Amalgavirus* genus, which currently has four determined species. In this study, by RNA-seq and data analysis, we determined three RNA viruses in the melon with an obvious yellow vein symptom, of which two viruses are the newly reported dsRNA viruses: one is in the *Partitiviridae* family, and the other is in the *Amalgaviridae* family.

The taxonomic reorganization of family *Partitiviridae* was ratified by the ICTV mainly based on phylogenetic results [4]. There are five clades in the phylogenetic trees constructed with the approved and probable species of partitiviruses, which corresponds to the five genera: *Alphapartititivirus*, *Betapartitivirus, Deltapartitivirus*, *Gammapartitivirus*, and *Cryspovirus.* In addition to the phylogenetics, several other demarcation criteria are also worthy of consideration. (i) The host range was used as a defining characteristic of each genus. *Alphapartitivirus* and *Betapartitivirus* consist of plant or fungal viruses; *Deltapartitivirus* includes only plant viruses; *Gammapartitivirus* includes only fungal viruses; and *Cryspovirus* includes only protozoal viruses. (ii) The genome segment and protein lengths are of a characteristic range for each genus [3]. (iii) Pairwise identity scores provide further evidence for genus demarcations. There are <24% RdRp aa sequence identities in pairwise comparisons of viruses from different genera [3]. (iv) Conserved 5′-terminal and 3′-terminal genomic sequences are present in different genera [33,34]. For instance, the consensus 5′-CGCAAAA-3′ sequence is in the 5′-terminus of *Gammapartitivirus*, the *Deltapartitivirus* have a conserved 5′-AGAAUUU-3′ sequence at its 5′-terminus, and the 3′-terminal regions of *Alphapartitivirus* and *Betapartitivirus* contain long poly (A) stretches [3,35]. With the characterization of the CmCV genome, we found that the CmCV possesses many common features of the genus *Deltapartitivirus*. Firstly, CmCV RdRp showed the highest identities with the RdRps of deltapartitiviruses, and several conserved motifs in deltapartitivirus RdRps were also present. Secondly, phylogenetic analyses of partitivirus RdRps and CPs place CmCV in the *Deltapartitivirus* clade with the other determined and probable deltapartitiviruses. Thirdly, pairwise identity scores aligned by MAFFT and SDT indicated that CmCV RdRp has the relatively higher identity within the deltapartitiviruses, compared to the other four genera, although the highest identity is only 60.1%. Lastly, the 5′-termini of both CmCV dsRNA-1 and dsRNA-2 contain characteristics of *Deltapartitivirus*, such as the conserved 5′-AGAAUUU-3′ sequence and predicted stem-loop structure in the 5′UTR. Although clearly a deltapartitivirus, CmCV is somewhat distinct from other species in this genus, because the length of its dsRNA-2 and its encoded CP, 1715 bp and 480 aa, respectively, is larger than those reported for other deltapartitiviruses (i.e., dsRNA-2 of 1415–1575 bp and CP of 337–430 aa). Collectively, these findings suggested that CmCV should be assigned to the genus *Deltapartitivirus* and considered as a candidate new species due to its low identity with other deltapartitiviruses.

Most of the putative amalgaviruses that have been identified recently were discovered by the analyses of a transcriptome dataset. Similarly, we identified CmAV1 by exploring the melon transcriptome. Phylogenetic tree construction and pairwise identity scores of RdRp demonstrated that CmAV1 is closely related to amalgaviruses, showing <57.7% identities with the other amalgaviruses, prompting us to propose CmAV1 as a new member of the genus *Amalgavirus*. The RdRps of the genus *Amalgavirus* are predicted to be expressed as ORF1+2 fusion proteins, which are generated through the +1 PRF mechanism using the typical UUU_CGN motif in most amalgaviruses. However, some amalgaviruses, such as *Capsicum annuum* amalgavirus and southern tomato virus, were found to not contain this conserved motif [12]. The UUU_CGN motif detected in the CmAV1 genome suggests that the CmAV1 RdRp can also be expressed by the +1 PRF mechanism. The analyses of the transcriptome dataset and semi-quantitive RT-PCR confirmed the existence of RNA1a, which implies that a shorter RdRp may be expressed without one of the conserved motifs among amalgaviral RdRps.

RNA recombination is one of the most important mechanisms performed by many animal and plant viruses to generate genomic diversity and adapt to complex environments or different hosts, among others functions [36]. Some recombination events are the crossovers between different viral sequences belonging to the same or different taxonomic groups, infecting different hosts, and with diverse geographical distribution [36,37]. The other recombination events exist amongst the same RNA templates by hopping over the RNA molecules in the process of replication producing subviral defective RNAs [38]; for example, the defective RNA of the tobacco mosaic virus genome with the deletion of certain internal sequences appeared to constitute a core region that had an influence on the efficiency of replication [39]. The identified four defective genomes of the influenza B virus, which contained obvious deletions in the central regions with each having the potential for encoding a novel polypeptide, could potently inhibit virus replication [40], etc. The analysis of the RNA structure of the deleted segment and sequence characteristics indicated that the generation of RNA1a may be the consequence of RNA recombination. In addition to the +1 PRF mechanisms, RNA recombination could be another way to broaden the protein diversity and regulate the expression level during the CmAV1 infection. If CmAV1 RNA1a is a product of RNA recombination, it would be the first reported defective-RNA for a plant-infecting dsRNA virus.

In most cases, the plants infected by deltapartitiviruses or amalgaviruses have been reported as asymptomatic, but there are exceptions. For example, the *Cannabis* cryptic virus was the sole virus found in symptomatic hemp plants showing streaking [9], while southern tomato virus was identified in tomato exhibiting interveinal chlorosis [18], and the blueberry latent virus was detected in bushes with fruit abortion [16,17]. However, there are no specific correlations between symptoms and these viruses.

MNSV, which was also identified in this study, is able to induce necrotic spots symptom in infected melon [20]. To test if MNSV induced the yellow vein symptom in the melon, mechanical inoculation of the sample was conducted on four melon cultivars, but no symptoms or MNSV were detected. This result may be due to the relatively low titers of MNSV in the samples.

In conclusion, we have identified two new dsRNA viruses and MNSV in the yellow vein melon leaves. However, the association between symptoms and these viruses could not be confirmed. Whether the yellow vein symptom was caused by a single virus or co-infection will require further investigation.

## Figures and Tables

**Table 1:** Summary of the RNA viruses identified in the symptomatic melon leaves. ORF: open reading frame.

Full Name	Abbrev.	Segment	Accession	Length	ORF	Position	Length (aa)
*Cucumis melon* cryptic virus	CmCV	RNA1	MH479772	1592 bp	RdRp	94–1527	477
		RNA2	MH479773	1715 bp	CP	97–1539	480
*Cucumis melon* amalgavirus 1	CmAV1	RNA1	MH479774	3424 bp	ORF1 (putative CP)	143–1297	384
					ORF1 + 2 (fusion protein, RdRp)	143–973, 975–3293	1049
		RNA1a			ORF1 + 2a	143–973, 975–2692,	1022
						2774–3293	
melon necrotic spot virus	MNSV	RNA1					

## Supplementary Materials

The following are available online at http://www.mdpi.com/1999-4915/11/1/81/s1. Figure S1: The secondary structure formed by the 5′UTR and 3′UTR of CmAV1. Table S1: Primers used for PCR amplification and RACE. Table S2: The GenBank accession number and abbreviation of the partitiviruses used in this study and amino acid sequences identities of CmCV with other partitiviruses. Table S3: The pairwise identity scores of RdRp and CP in the family of *Partitiviridae* aligned by MAFFT software. Table S4: The abbreviation of the amalgaviruses used in this study and putative CP sequence identities of CmAV1 with other amalgaviruses. Table S5: The pairwise identity scores of RdRp in the family of *Amalgaviridae* aligned by MAFFT software.
